# Holmes Tremor Secondary to a Brainstem Haemorrhage With Partial Symptomatic Improvement Following Levetiracetam Use

**DOI:** 10.7759/cureus.104226

**Published:** 2026-02-25

**Authors:** Neil P Lodhia, Caoilin Marstrand, Paul Bolaji

**Affiliations:** 1 Medicine, Dorset County Hospital, Dorchester, GBR; 2 Stroke Medicine, Dorset County Hospital, Dorchester, GBR

**Keywords:** brain stem stroke, holmes tremor, stroke, stroke rehab, tremor management

## Abstract

Holmes tremor is a rare movement disorder linked to brainstem pathology and characterised by a combination of rest, postural, and intention tremors. These tremors are typically low frequency (<5 Hz), high amplitude, and often debilitating, significantly impairing rehabilitation and functional recovery.

We present a man in his 30s with a right-sided brainstem haemorrhage of unknown aetiology who developed a delayed-onset, left-sided proximal upper limb tremor approximately one month after the initial insult. The tremor was initially attributed to anxiety and neuropathic pain, and treatment with gabapentin was ineffective. On specialist review, the tremor was identified as a high-amplitude, irregular, low-frequency 3 Hz tremor occurring at rest and exacerbated by posture and action, consistent with Holmes tremor.

The patient was treated with titrated levetiracetam, resulting in partial but clinically meaningful symptomatic improvement, enabling greater participation in neurorehabilitation and discharge to a level 2a neurorehabilitation facility. Follow-up magnetic resonance imaging demonstrated hypertrophic olivary degeneration, supporting the diagnosis. Although tremor persisted and remained functionally intrusive, this case highlights the clinical features, underlying pathophysiology, and potential role of levetiracetam as a therapeutic option in the management of post-stroke Holmes tremor.

## Introduction

Movement disorders are recognised complications of stroke and are broadly classified as either hyperkinetic or hypokinetic. A study of 1,500 stroke patients found that 3.7% developed an acute or delayed movement disorder [1]. However, another study by Ghika-Schmid et al. [2] reported a lower incidence of 1% in 2,500 patients. Alarcón et al. [1] also observed that post-stroke movement disorders are more common in patients with haemorrhages involving the basal ganglia, thalamus, or brainstem, as seen in our patient.

In a review of 284 published cases of post-stroke movement disorders, tremors occurred in approximately one in six patients, occurring mostly in the haemorrhagic stroke subtype [3]. In a prospective observational study, Samra et al. [4] reported that 2.2% of stroke patients developed intention tremors or ataxia. This accounted for more than 50% of post-stroke movement disorders. Other tremor types, including Holmes tremor, were seen in 15% of the patients.

Holmes tremor, first described by Gordon Holmes in 1904, is a rare movement disorder characterised by a combination of rest and intention tremors, often with a postural component. These tremors are typically irregular, coarse, and high amplitude, with a frequency of less than 4.5 Hz [5,6]. They are often associated with additional neurological features such as dystonia, choreoathetosis, ataxia, and hypoesthesia, with varying proximal or distal tremor predominance [7-9]. Onset is classically delayed, occurring weeks to months after a structural brain lesion. Medical management is often challenging, with variable responses to pharmacological therapies and frequent consideration of surgical options in refractory cases [7-9].

This case highlights the importance of careful phenomenological assessment of post-stroke tremor, the relevance of delayed-onset Holmes tremor following brainstem haemorrhage, and the potential for partial symptomatic benefit with levetiracetam, facilitating functional rehabilitation.

## Case presentation

A previously healthy man in his 30s presented with sudden-onset headache, vomiting, slurred speech, and confusion. On examination, he exhibited diplopia, bidirectional nystagmus, right abducens palsy, left upper motor neuron facial palsy, left upper and lower limb weakness (more pronounced proximally), reduced sensation in the left upper limb, and dysarthria.

A non-contrast CT scan of the head revealed a right-sided brainstem haemorrhage involving the pontine region, close to the middle cerebellar peduncle. CT angiography and digital subtraction angiography did not identify an underlying vascular malformation. The patient was found to be hypertensive and was managed using the ABC stroke protocol (A: rapid reversal of anticoagulation; B: blood pressure control; C: care pathway, prompt referral to the neurosurgeons), which included strict blood pressure control and neurosurgical evaluation.

Approximately one month into his admission, the patient developed a coarse, intermittent left arm tremor affecting the left upper limb. The tremor was present at rest and became more pronounced during sustained and goal-directed movement, particularly during physiotherapy sessions. Initially, this was attributed to anxiety or pain-related phenomena. However, the tremor’s proximal distribution, irregular pattern, and high amplitude, clinically estimated at 3 Hz, significantly interfered with his rehabilitation. At this stage, he was reliant on a hoist for transfers and could only perform limited upper limb exercises.

A trial of gabapentin (up to 300 mg three times a day) was undertaken sequentially without meaningful improvement in the tremor but aided in the patient’s neuropathic pain. The patient preferred not taking anxiolytics. On specialist stroke review, the tremor was recognised as fulfilling the criteria for Holmes tremor, given the phenomenology and the underlying brainstem pathology.

Investigations

**Figure 1:**
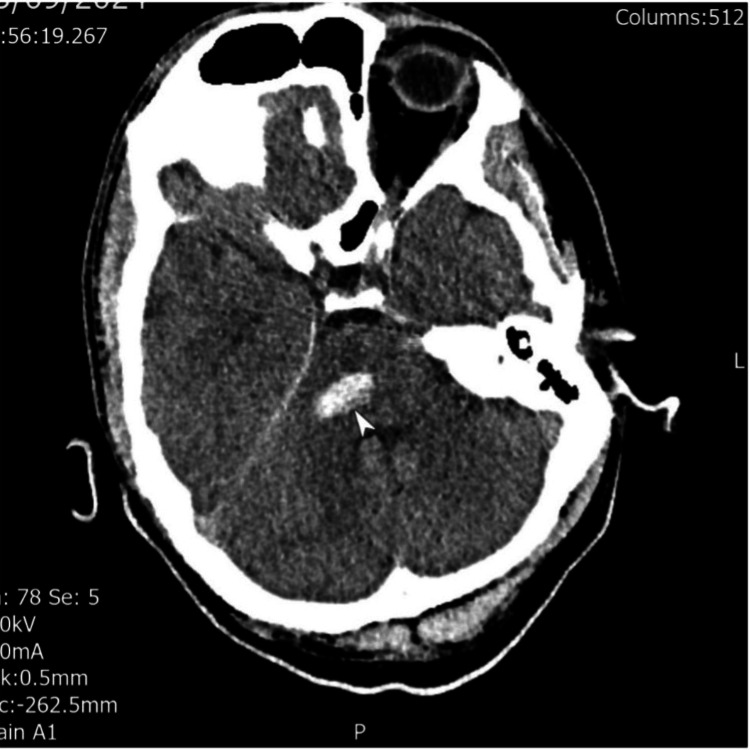
Admission CT brain-axial showing a right pontine bleed close to the middle cerebellar peduncle

**Figure 2:**
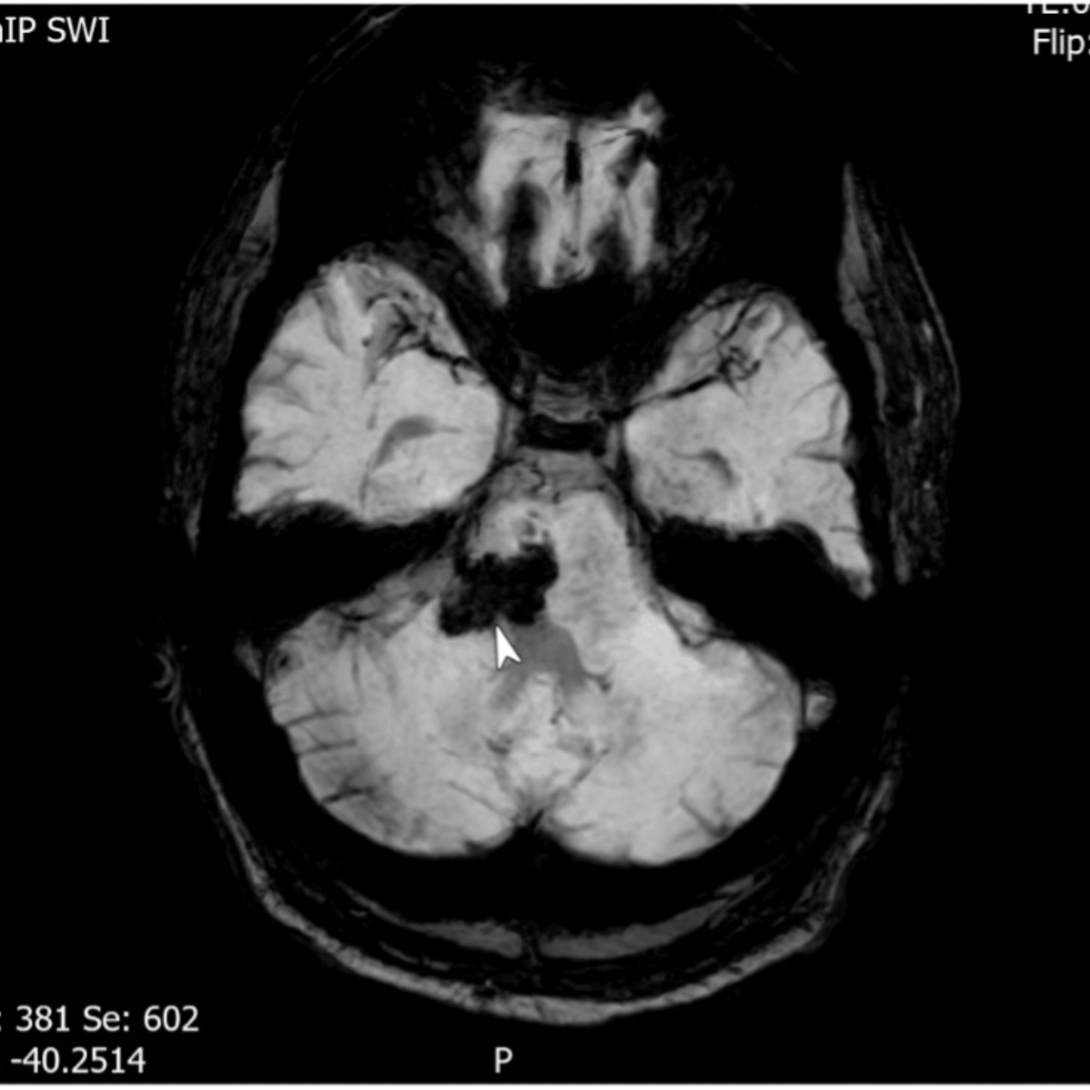
Axial SWI (susceptibility weighted imaging) brain MRI (six weeks after presentation) showing the old right pontine bleed adjacent to the middle cerebellar peduncle

**Figure 3:**
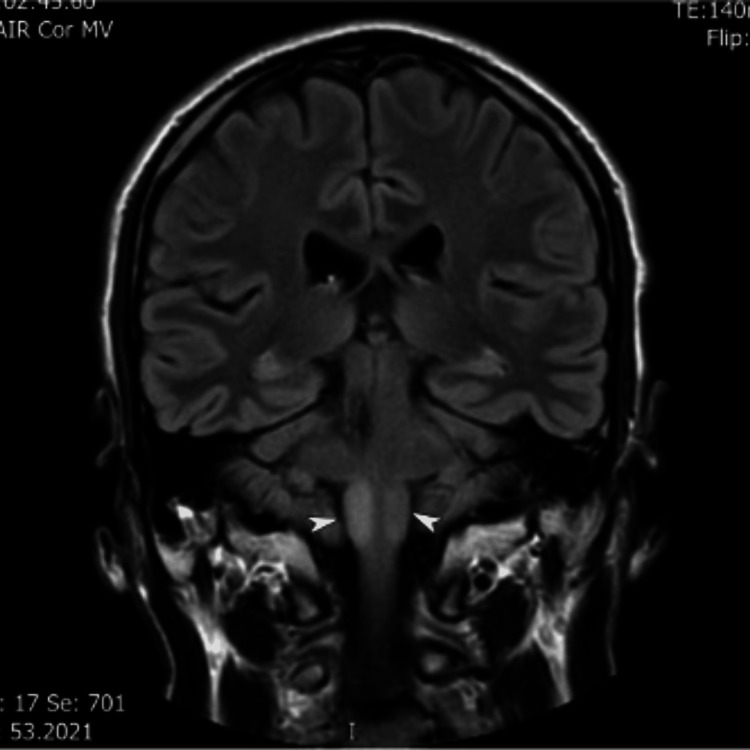
Coronal FLAIR (Fluid Attenuated Inversion and Recovery) MRI (six months after presentation) showing bilateral hypertrophic degeneration of olivary nuclei, more conspicuous on the right

The patient’s electrocardiography and routine blood tests were unremarkable. Neuroimaging findings were as follows. CT of the head at admission demonstrated right middle cerebellar peduncle haemorrhage with perilesional oedema (Figure 1). MRI and MRA (six weeks post-stroke) demonstrated no vascular malformation, infarction, or space-occupying lesion. A small haemosiderin-lined cavity was consistent with prior haemorrhage (Figure 2). MRI (six months post-stroke) showed high signal and swelling within the medulla bilaterally, consistent with hypertrophic olivary degeneration, further supporting the diagnosis of Holmes tremor (Figure 3). 

Differential diagnosis

Early in the clinical course, other hyperkinetic movement disorders were considered. These included anxiety-related physiological tremor, cerebellar intention tremor, vascular parkinsonism, and choreoathetosis. Choreoathetosis was briefly considered due to the coarse, involuntary nature of the movements. However, it was later excluded because the patient’s movement was rhythmic and tremulous, present at rest, in posture, and in action, rather than flowing or writhing.

The combination of delayed onset, proximal predominance, low-frequency, high-amplitude tremor with rest, postural, and intention components, and supportive neuroimaging findings favoured a diagnosis of Holmes tremor.

Treatment and outcome

The patient was commenced on levetiracetam, which has shown efficacy in Holmes tremor [10,11]. He was initially started on 500 mg twice daily for nine days, followed by an increase to 750 mg in the morning and 500 mg in the evening, which was then continued. This led to partial symptomatic improvement, with a reduction in tremor amplitude and improved endurance, with the tremor frequency remaining largely unchanged. The result was greater engagement in neurorehabilitation.

The patient was discharged to a level 2A specialist rehabilitation facility for approximately four months, where he underwent intensive multidisciplinary rehabilitation for five months. Level 2A facilities provide local-level specialist inpatient facilities for medically stable patients with complex neurological needs and requiring a high level of staff input. Levetiracetam was increased to 750 mg twice a day and gabapentin to 700 mg three times a day. A trial of Co-Beneldopa was also undertaken without additional benefit and was discontinued.

Given the persistence of functionally limiting tremor despite optimised medical therapy, the patient was referred to a tertiary neurosurgical centre for consideration of deep brain stimulation (DBS) following outpatient review, approximately three months into admission in the rehabilitation facility. He continues to receive outpatient neurorehabilitation and spasticity management (Table 1).

**Table 1:** Timeline of the patient's journey, starting from Week 0 at the index presentation to Week 33 where patient was discharged from the neurorehabillitation centre

Timepoint	Key events	Investigations	Management
Day 0	Sudden onset headache, vomiting, slurred speech, and confusion	CT of the head: right middle cerebellar peduncle haemorrhage with perilesional oedema	Blood pressure control and neurosurgical review (ABC stroke protocol)
Weeks 4-6	New left upper limb tremor (rest, postural, intention; proximal, high amplitude, ~3 Hz)	MRI of the brain: a small haemosiderin-lined cavity was consistent with prior haemorrhage	Trial of gabapentin with no benefit to the tremor
Weeks 7-9	Tremor recognised as Holmes tremor		Levetiracetam commenced at 500 mg twice daily initially and then increased to 750 mg and 500 mg. This showed some improvement
Weeks 10-33	Discharge from acute hospital to Level 2A Rehabilitation centre at week 10	MRI/MRA showing bilateral hypertrophic degeneration of olivary nuclei, more conspicuous on the right at week 24	Levetiracetam increased to 750 mg BD. Co-Benelodpa trialled without success. Referral for deep brain stimulation
Week 33	Discharge from the Neurorehabilitation centre		

## Discussion

Holmes tremor, also called rubral, cerebellar outflow, or midbrain tremor, usually presents with tremor at rest that worsens with action and intention [12]. Holmes tremor is a rare post-lesional tremor syndrome characterised by a combination of rest, postural, and intention tremors, typically involving the upper limb and more severe proximally [13]. Our patient presented similarly, with left-sided proximal limb resting and intention tremor that worsened with posture, especially during physiotherapy sessions.

The features of Holmes tremor include (1) a unilateral resting, intention, and postural tremor; (2) more severe proximally than distally; (3) onset within a few weeks to two years after the initial lesion; and (4) evidence supported by MRI imaging [8,14].

The aetiology of Holmes tremor includes vascular causes, ischaemic stroke and haemorrhagic stroke, which form the majority, as well as head trauma and neurodegenerative diseases [7,8,9,15]. Patients also tend to present with a delayed onset of tremor following the initial insult, typically over a period of two months [9,11,15].

Our patient’s presentation was in line with this, as he developed a tremor one month into admission. His neuroimaging showed a right pontine bleed with evidence of hypertrophic olivary degeneration bilaterally in the brainstem, further supporting the diagnosis.

The pathophysiology of Holmes tremor has often been debated in the literature. Disruption of the circuit connecting the cerebellum, thalamus, red nucleus, globus pallidus pars interna, and the pontomedullary junction has been implicated in patients with Holmes tremor by MRI [11,14]. It is theorised that haemorrhage, ischaemia, infection, or neoplasm in the midbrain may disrupt multiple pathways, including the dopaminergic nigrostriatal, dentatorubro-olivary, and cerebellothalamic circuits [8,11,14]. Several authors also suggest that multiple lesions may be required for symptoms to manifest as Holmes tremor [7,16,17].

In our index patient, MRI brain completed at six months after his haemorrhage showed bilateral hypertrophic olivary degeneration. This supports involvement of the dentato-rubro-olivary network and provides radiological corroboration of the clinical diagnosis in this report. The superior and inferior olivary nuclei are located at the junction of the medulla oblongata and pons, where they act as relay centres integrating motor and sensory input to the cerebellum. They provide corrective error signals to cerebellar circuits, thereby fine-tuning movement and suppressing unwanted activity. They have been linked to movement disorders such as multiple system atrophy and Holmes tremor [18].

Due to the heterogeneity of clinical symptoms and imaging findings reported in the literature, no single neurological localisation has been determined for Holmes tremor. For instance, Joutsa et al. [19] discuss that lesions associated with Holmes tremor may localise to an alternative main brain circuit involving the cerebellum, globus pallidus pars interna (GPi), thalamus, red nucleus, and pontomedullary junction [19]. Such widespread involvement could lead to a combination of resting, postural, and intention tremors, as both dopaminergic and non-dopaminergic pathways are affected, and may explain why medical and surgical interventions often lead to variable responses [9,14,20].

Medical management of Holmes tremor remains challenging, with heterogeneous responses reported for dopaminergic agents, anticholinergics, beta-blockers, anticonvulsants, and other therapies. Levetiracetam has been reported in isolated cases to provide benefit, possibly through modulation of excitatory and inhibitory synaptic transmission [7,9,11,17,21]. Our case report contributes to the limited literature on the use of levetiracetam in Holmes tremor, although with partial response [22].

Levetiracetam, primarily used as an anti-epileptic medication [23], has seen its application extend to treating a variety of neurological conditions, including migraines, neuropathic pain, and essential tremors [23-25]. Its mechanism of action is not completely understood, but it is believed to act on synaptic vesicle protein 2A (SV2A), downregulating glutamate activity in excitatory synapses and enhancing GABA release in inhibitory synapses [23,24]. This modulation may provide an explanation for its therapeutic role in managing a range of neurological disorders.

In our patient, levetiracetam resulted in partial but clinically meaningful improvement, facilitating engagement in rehabilitation, although tremor was not abolished. The degeneration of the olivary nuclei, shown on MRI, could explain the partial response despite the initial improvement with levetiracetam.

Other non-pharmacological treatments for Holmes tremor include DBS. DBS is generally reserved for medically refractory cases and may offer benefit depending on target selection and underlying network disruption. In this patient, DBS was considered due to persistent functional disability despite optimised pharmacological therapy rather than failure of levetiracetam.

DBS has shown response rates of 31-57.8%, depending on the targeted area [14,20]. DBS to the GPi has demonstrated better clinical improvement in postural tremor, with long-lasting benefits, compared with medical treatment, although its effects on resting and intention tremors are similar [14,20].

While brainstem haemorrhage is a recognised cause of Holmes tremor, this case is notable for the combination of delayed onset, detailed phenomenological characterisation, radiological confirmation of hypertrophic olivary degeneration, and partial response to levetiracetam.

## Conclusions

Holmes tremor is an uncommon but important cause of delayed post-stroke movement disorder, particularly following brainstem lesions. Accurate diagnosis relies on careful clinical characterisation of tremor phenomenology, including distribution, frequency, amplitude, and its relationship to rest, posture, and action, supported by targeted neuroimaging.

Medications such as levetiracetam might improve symptoms in patients with Holmes tremor; however, patients might need to consider surgical therapy such as DBS if tremor is intractable despite several trials of medications with varying mechanisms of action.
